# Effect of Tooth Types on the Accuracy of Dental 3D Scanners: An In Vitro Study

**DOI:** 10.3390/ma13071744

**Published:** 2020-04-09

**Authors:** Keunbada Son, Kyu-bok Lee

**Affiliations:** 1 Department of Dental Science, Graduate School, Kyungpook National University, Daegu 41940, Korea; sonkeunbada@gmail.com; 2 Advanced Dental Device Development Institute, Kyungpook National University, Daegu 41940, Korea; 3 Department of Prosthodontics, School of Dentistry, Kyungpook National University, Daegu 41940, Korea

**Keywords:** dental 3D scanner, accuracy, desktop scanner, intraoral scanner, tooth type

## Abstract

The purpose of this study was to evaluate the accuracy of dental three-dimensional (3D) scanners according to the types of teeth. A computer-aided design (CAD) reference model (CRM) was obtained by scanning the reference typodont model using a high-precision industrial scanner (Solutionix C500, MEDIT). In addition, a CAD test model (CTM) was obtained using seven types of dental 3D scanners (desktop scanners (E1 and DOF Freedom HD) and intraoral scanners (CS3500, CS3600, Trios2, Trios3, and i500)). The 3D inspection software (Geomagic control X, 3DSystems) was used to segment the CRM according to the types of teeth and to superimpose the CTM based on the segmented teeth. The 3D accuracy of the scanner was then analyzed according to the types of teeth. One-way analysis of variance (ANOVA) was used to compare the differences according to the types of teeth in statistical analysis, and the Tukey HSD test was used for post hoc testing (α = 0.05). Both desktop and intraoral scanners showed significant differences in accuracy according to the types of teeth (*P* < 0.001), and the accuracy of intraoral scanners tended to get worse from anterior to posterior. Therefore, when scanning a complete arch using an intraoral scanner, the clinician should consider the tendency for the accuracy to decrease from anterior to posterior.

## 1. Introduction

The introduction of dental computer-aided design and computer-aided manufacturing (CAD-CAM) has enabled the use of a digital workflow instead of a conventional workflow that relies on operator experience and skills [1,2,3,4,5]. The first digital workflow was partially digital and used a desktop scanner accompanied by impression taking and the fabrication of a working model [6,7,8,9]. Since then, the introduction of intraoral scanners in the 1980s has enabled a fully digital workflow [1,2,3,10]. This has reduced the effects of the operator’s experience and possible errors in the material (impression and dental stone) [3,7,8,11,12]. Advances in digital workflows have made it possible to evaluate temporomandibular joint (TMJ)-related diseases through cone-beam-computed tomography (CBCT) scans [13,14]. Therefore, a recent study reported better accuracy in digital workflows than conventional workflows [15]. Furthermore, digital workflow is being verified as a reliable alternative in clinical practice through clinical trials [16,17,18].

The intraoral scanner has emerged as a chairside concept for fixed prosthetic treatment [19,20,21,22]. However, it is now being applied to many other treatment procedures, including removable prosthetic treatments, orthodontic treatments, and implant planning procedures [10,23,24,25]. The ability to scan the teeth and soft tissues and perform three-dimensional (3D) modeling in a faster and more convenient manner than conventional workflows indicates a potential for wider applications [23,24,25]. In addition, 3D modeling obtained from the oral cavity is not only used at the chairside but may also play an important role in communications between dentist, dental technician, and patient [10,26,27].

Recent studies have evaluated the accuracy of intraoral scanners in various ways [20,21,22,28,29,30,31,32]. A 3D analysis has been performed by measuring distances using a datum point or shape or through the overlap of the CAD Reference Model (CRM) and CAD Test Model (CTM) [1,5,8,12,19,27,33,34]. The CRM is acquired with a high-resolution scanner, such as an industrial 3D scanner [2,5,12,27,33,34,35]. Using 3D inspection software, the CTM and CRM overlap to the optimal (as close as possible) location, and the root-mean-square (RMS) is calculated from the average of the distances of all the point clouds in the CRM and CTM [2,5,12,23,29,30,31]. This RMS has become an evaluation criterion for the accuracy of scanned data in many studies [1,2,5,8,10,17,27,33,34,35]. Previous studies have suggested that the final restoration may be incorrectly fitted if the scanning accuracy exceeds 100 µm [5,11,19]. In addition, due to the acceptable cement space of the prosthesis, an accuracy-tolerance range of less than 100 µm is preferred [5,11,19]. However, since the resin cement thickness over 50 µm tends to crack [36], the accuracy-tolerance range of the scanner that is less than 50 µm should be considered.

The limited intraoral conditions, the impact of the scanning accuracy on the prosthesis, and the impact of the increase of the scanning range on the accuracy have been investigated for the development and application of intraoral scanners [5,27,34]. Previous studies have shown that different complete arch forms of patients may affect the accuracy of intraoral scanners [19,33]. Some previous studies have also reported that the accuracy of intraoral scanners is inadequate for complete or edentulous arches [5,8], while others have reported higher accuracy compared to conventional impressions [19,20]. Apart from the accuracy of scanning, the disadvantages due to limited space; the wet environment; the expense; and the maintenance cost of the intraoral scanner still exist [37]. There are many studies on the accuracy of intraoral scanners under various conditions [1,2,5,8,10,17,27,33,34,35], and studies on complete-arch scans are required for various types of scanners [5,11,19]. The size, shape, and location of teeth vary in the oral cavity [38]. However, studies comparing the accuracy of different types of teeth located in the complete arch are lacking.

Therefore, the purpose of this study is to evaluate the accuracy of 3D scanners according to the types of teeth. The null hypothesis of this study is that the accuracy of 3D scanners does not vary by tooth type.

## 2. Materials and Methods 

A pilot experiment was conducted five times using a power analysis software (G*Power v3.1.9.2, Heinrich–Heine–Universität, Düsseldorf, Germany) [5] to determine the sample size as 15 (actual power=99.93%; power=99.9%; α = 0.05). This finding indicated that the proposed study required a minimum of 15 subjects to ensure a power > 99.9%.

**Figure 1:**
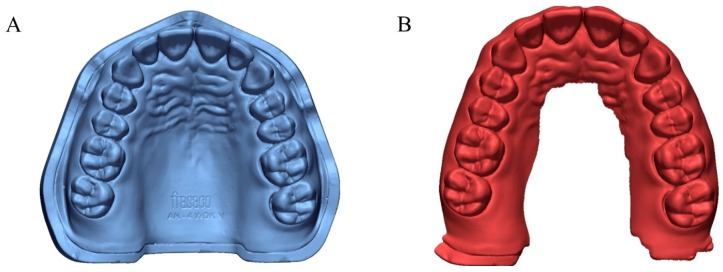
Virtual models. (**A**) CAD reference model (CRM). (**B**) CAD test model (CTM).

A typodont model (ANKA-4 V CER, Frasaco GmbH, Tettnang, Germany) was scanned using an industrial 3D scanner (Solutionix C500, MEDIT, Seoul, Republic of Korea) with a resolution of 2 × 5 Mpx and Blue LED, and high-resolution CRM files were obtained by five scans (Figure 1A). A high-resolution CRM file was obtained by merging five CRMs acquired through an industrial 3D scanner. The industrial 3D scanner from the manufacturer verified an accuracy below 10 µm.

**Figure 2:**
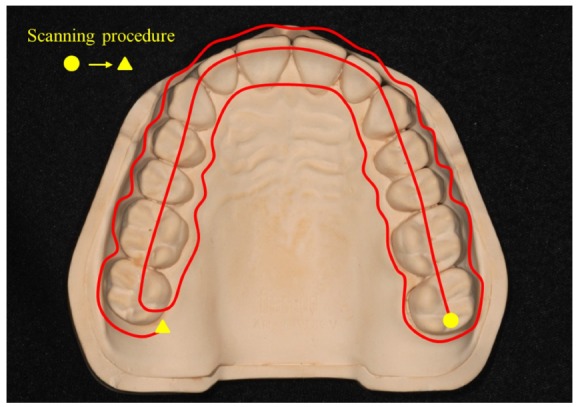
Strategies of complete arch scanning.

Five intraoral scanners (CS3500 (Carestream Dental, Atlanta, GA, USA), CS3600 (Carestream Dental), Trios2 (3Shape, Copenhagen, Denmark), Trios3 (3Shape, Copenhagen, Denmark), and i500 (MEDIT, Seoul, Republic of Korea)) and two desktop scanners (E1 (3Shape, Copenhagen, Denmark) and DOF Freedom HD (DOF, Seoul, Republic of Korea)) were used to obtain the CTM (Figure 1B). The scan sequence was determined according to the manufacturer’s instructions for the complete scan and from the published literature on scan strategies for optimal results (Figure 2). In accordance with ISO-12836, each scanner scanned at an ambient temperature of 23 ± 2 °C and humidity of 50 ± 5% considering the oral environment, and one operator (K.S.) skillful in the use of each intraoral and desktop scanner performed the scanning 15 times [39,40,41]. The standard tessellation language (STL) files were subsequently obtained for 3D analysis.

Since a variety of 3D inspection software are aligned using different protocols, 3D analysis results may differ. Thus, Geomagic’s 3D inspection software (release 2018.0.0, Geomagic control X, 3D Systems, Rock Hill, SC, USA) recommended by ISO-12836 was used in the present study [39]. 

**Figure 3:**
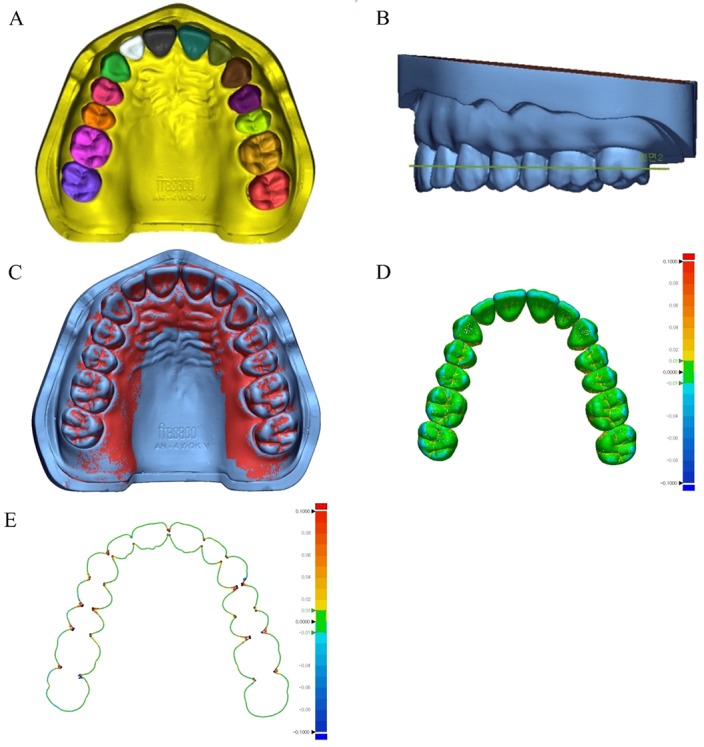
Three-dimension (3D) analysis procedure. (**A**) Tooth segmentation, (**B**) virtual plane for horizontal displacement, (**C**) superimposition of CRM and CTM, (**D**) 3D displacement, and (**E**) horizontal displacement.

First, for comparison of the 3D displacement according to the tooth types, the CRM file was segmented into each tooth (Figure 3A). To evaluate the horizontal displacement of the dentition, a plane based on a hypothetical line connecting the central incisor and half of the second molar in the sagittal plane was formed (Figure 3B). After the CRM file was prepared, the CTM file was retrieved and the initial alignment was conducted. They were superimposed by the best fit alignment based on all the segmented teeth (Figure 3C). The sampling rate was set at 100%.

The 3D differences between CRM and CTM were calculated for all data-point clouds of the segmented teeth. The data point was calculated by RMS value, and the following Equation(1) was used [3,5,10,12,33]:

(1) $$RMS=\frac{1}{\sqrt{n}}\times \sqrt{\overset{n}{\underset{i=1}{\sum }}{\left(X_{1,i}-X_{2,i}\right)}^{2}}$$

where X_1,*i*_ is the measurement point of *i* of the CRM, X_2,*i*_ is the measurement point of *i* of the CTM, and *n* is the number of all points measured in each analysis.

The RMS value shows how the two different sets of data deviate from zero [5]. A low RMS value indicates a good 3D agreement of the superimposed data. Each 3D comparison is shown as a color difference map, having a range of ± 100 µm (20 color segments) and a tolerance range (green) of ± 10 µm (Figure 3D). The red region (10 µm to 100 µm) indicates that the CTM data are located above the CRM data, implying that the scanning is larger than the reference data. The blue region (−10 µm to −100 µm) indicates that the CTM data are located below the CRM data, implying that the scanning is smaller than the reference data. The green region (±10 µm) corresponds to areas that were scanned precisely. The horizontal displacement was also calculated as RMS in the hypothetical plane (Figure 3E).

All data were analyzed using statistical software (SPSS, release 25.0, IBM, Chicago, Illinois, USA) (α = 0.05). First, the normal distribution of data was investigated through the Shapiro–Wilk test. As a result, the differences between the groups were analyzed using one-way ANOVA and Tukey HSD test as post hoc.

## 3. Results

**Figure 4:**
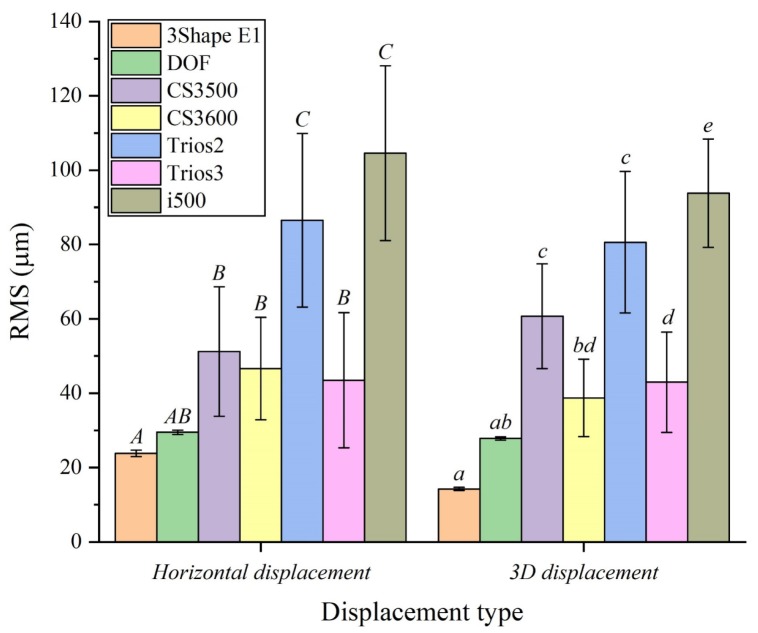
Horizontal displacement and 3D displacement according to the 3D scanners. Different letters indicate that the difference between the groups is significant by Tukey’s HSD post hoc testing (*P* < 0.05).

Both horizontal displacement and 3D displacement showed significant differences in accuracy depending on the type of 3D scanner (*P* < 0.001) (Table 1 and Figure 4). Excellent accuracy was achieved in the desktop scanner group (< 30 µm), and the intraoral scanners did not exceed 100 µm, except for the i500 group (104.6 ± 23.5 µm) (Table 1 and Figure 4).

**Figure 5:**
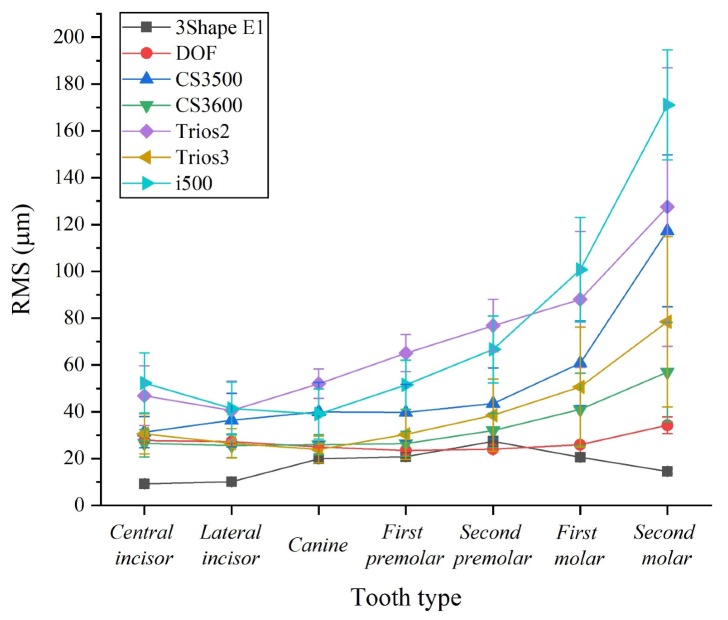
Comparison of the accuracy of 3D scanners according to the tooth type.

Both desktop and intraoral scanners had significant differences in accuracy according to the types of teeth (*P* < 0.001) (Table 2 and Figure 5). The intraoral scanner group tended to show worse accuracy with the second molar (Figure 5). On the other hand, the accuracy of the desktop scanner group did not tend to get worse towards the second molar and there was a little deviation (Figure 5).

**Figure 6:**
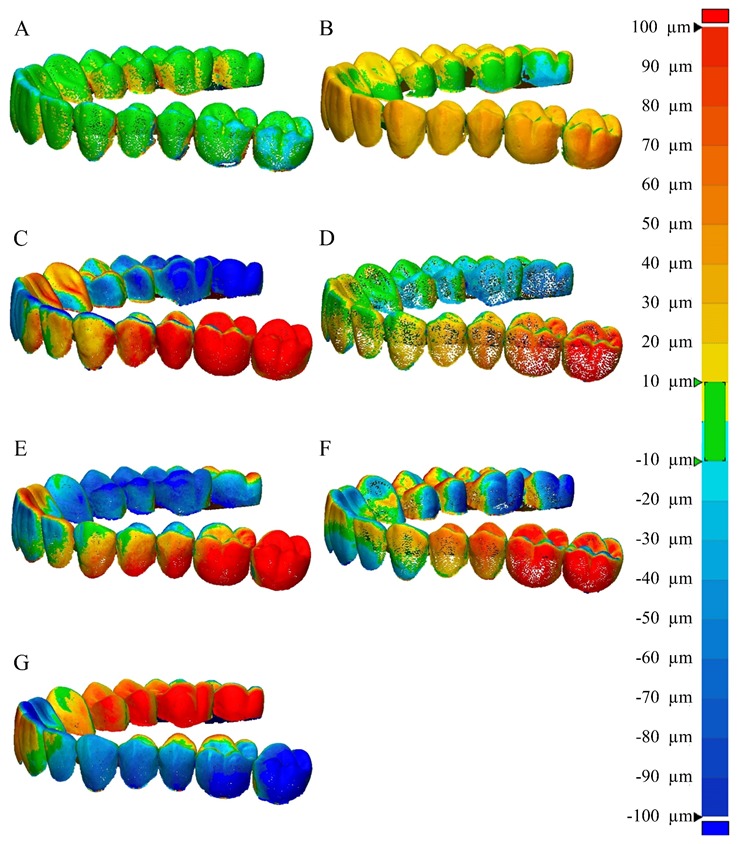
Comparison of the color map of 3D displacement according to the 3D scanners. (**A**) 3Shape E1, (**B**) DOF, (**C**) CS3500, (**D**) CS3600, (**E**) Trios2, (**F**) Trios3, and (**G**) i500.

The color difference map showed the largest green region (± 10 µm) in the E1 desktop scanner (Figure 6A), but all intraoral scanners had wide red (10 µm to 100 µm) and blue (−10 µm to −100 µm) regions (Figure 6).

**Figure 7:**
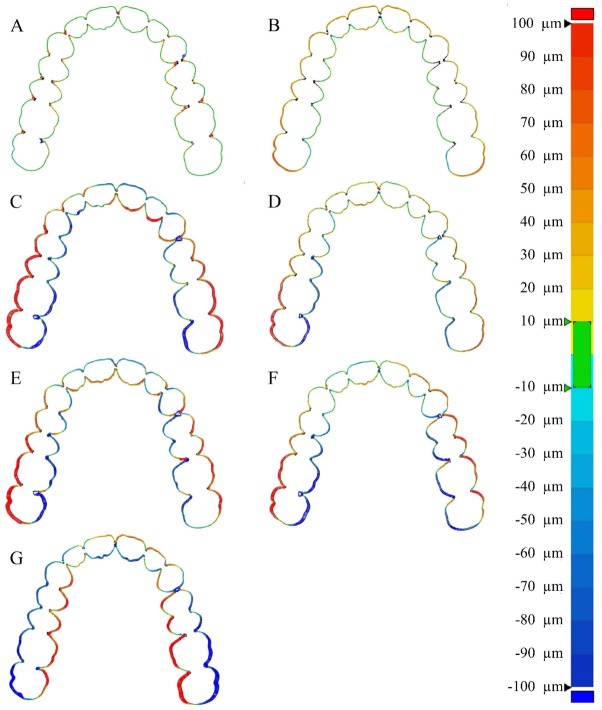
Comparison of the color map of horizontal displacement according to the 3D scanners. (**A**) 3Shape E1, (**B**) DOF, (**C**) CS3500, (**D**) CS3600, (**E**) Trios2, (**F**) Trios3, and (**G**) i500.

The desktop scanner group showed little horizontal displacement (Figure 7A,B), while all intraoral scanners, except for the i500 group, showed a horizontal displacement in the buccal direction towards the posterior region (Figure 7). In the i500 group, lateral displacement appeared lingually (Figure 7G).

## 4. Discussion

The purpose of this study was to evaluate the accuracy of 3D scanners according to the types of teeth. For this purpose, the null hypothesis was no difference in the scanning accuracy according to the types of teeth. However, the null hypothesis was rejected based on the results of the present study (*P* < 0.001) (Table 1 and Table 2), and it was concluded that the accuracy of 3D scanners is affected by the tooth type.

Ender et al. [19] analyzed 15 virtual models acquired by each intraoral scanner in the clinical environment, and Flügge et al. [32] analyzed 10 digital models acquired in the clinical environment, the findings of which suggest that intraoral conditions (saliva, limited intervals) contribute to scan inaccuracy [19,32]. However, the present study was not evaluated in the clinical environment but was evaluated using a reference model in an in vitro environment; it did not consider errors that may occur in the clinical environment. Therefore, further research is needed in the actual clinical environment.

What makes this study different from other studies is the process of 3D analysis. Lim et al. [21] and Jeong et al. [20] compared the scanning accuracy using intraoral scanners and analyzed the 3D accuracy of the complete arch by best fit alignment. Thus, the accuracy of the overall complete arch can be verified, but assuming that there is a large mismatch in one region, the best fit alignment process can cause errors in another region [5]. The 3D analysis in the present study was performed by segmenting the teeth according to the type of each tooth (Figure 3A). The best fit alignment of each tooth was performed to compare the displacements according to the types of teeth (Figure 3C). The segmented and best fit alignment of each tooth allowed us to compare the discrepancies between each tooth (Figure 3D,E).

The TMJ evaluation through CBCT in digital workflows accurately assesses the morphological symmetry of any anatomical structure with progress in radiographic techniques, and the use of sophisticated reverse engineering software products [13,14]. Therefore, accurate TMJ evaluation through CBCT can improve the quality of life in patients [13,14].

Ender et al. [19] suggested that the final restoration could be incorrectly fitted if the scan accuracy was more than 100 µm. Fukazawa et al. [11] suggested a tolerance of less than 100 μm because of the acceptable cement space for the prosthesis. In the present study, all tooth types in the desktop scanner group had an acceptable accuracy of less than 100 µm. In contrast, the CS3500 (second molar: 117.3 ± 32.3 µm), Trios2 (second molar: 127.5 ± 59.4 µm), and i500 (first molar: 100.7 ± 22.2 µm, second molar: 171 ± 23.4 µm) of the intraoral scanner group exceeded the clinically acceptable accuracy (Table 1 and Table 2). Thus, based on these results, the clinician should consider these errors when scanning the first and second molar using an intraoral scanner.

There have been many previous studies evaluating the accuracy of 3D scanners [1,2,5,8,11,12,29,42]. Park et al. [5] evaluated the accuracy using a desktop scanner (DOF) and intraoral scanners (Trios 2, Trios 3, CS3500, and CS3600), which were found to be 47.5 ± 1.6 µm, 343.4 ± 56.4 µm, 183.9 ± 49.7 µm, 209.9 ± 53.7 µm, and 118.9 ± 42.1 µm, respectively [5]. The poor accuracy compared to the results presented in this study (27.8 ± 0.4 µm, 80.6 ± 19 µm, 42.9 ± 13.4 µm, 60.7 ± 14 µm, and 38.7 ± 10.3 µm, respectively) (Table 1 and Table 2) is due to the difference in alignment between the CRM and CTM. Park et al. [5] confirmed the arch distortion by aligning only the tooth at which the scan was initiated, while the present study evaluated the accuracy by segmenting according to the type of tooth and aligning the entire tooth only. Michael Braian et al. [8] evaluated the accuracy using five intraoral scanners (Omnicam Sirona (Sirona Dental Systems, Bensheim, Germany), Itero Element (Align Technologies, San Jose, Calif, USA), Planmeca Emerald (Planmeca, Helsinki, Finland), CS3600, and TRIOS3), which were found to be <193 µm. Scanning of the complete arch using an intraoral scanner was not recommended [8]. The results of the present study ranged from 23.8 to 171 µm, depending on the position in the dental arch. Except for the CS3500 (second molar: 117.3 ± 32.3 µm), Trios2 (second molar: 127.5 ± 59.4 µm), and i500 (first molar: 100.7 ± 22.2 µm), the CS3600 and Trios3 were recommended for all tooth types. The difference in these results is due to differences in the accuracy evaluation method. Michael Braian et al. [8] analyzed the accuracy through distance error, but in this study, the accuracy was evaluated by 3D analysis using inspection software.

Compared to the intraoral scanner group, the desktop scanner group showed little horizontal displacement (Figure 7A,B). In contrast, all intraoral scanners, except the i500 group, showed a horizontal displacement in the buccal direction towards the posterior region (Figure 7C,D–F). In the i500 group, lateral displacements were shown in the lingual direction (Figure 7G). Intraoral scanners cause a horizontal displacement, because they transmit a light source from the end of a small head that can enter the oral cavity to the teeth of the complete arch and stitch the scanned area through a small scan range. Therefore, the aspects of horizontal displacement shown in this study will provide important data for manufacturers to develop better intraoral scanners.

There were some limitations to the present study. Previous studies have reported that the type of scanner can affect the marginal and internal fit of the prosthesis [43,44,45,46,47]. Depending on the results of the present in vitro study, there were few displacements of scanning in the desktop scanners, so further studies that consider the clinical environment are needed for the intraoral scanner. In the case of desktop scanners, the accuracy depends on impression material, so the problem is wider and concerns impression materials and teeth biomechanics during compression. Since only the scanning accuracy was evaluated in the present study, further research is needed to determine how the displacement of scanning affects the marginal and internal fit of the prosthesis when the actual prosthesis is fabricated.

The tooth shapes (steep inclination of cusps and depth of fissures, flat teeth) might have an influence on the scanner accuracy. Park et al. reported that complex structures could adversely affect scan accuracy [48]. Further study is needed on the effect of tooth shapes on scanner accuracy.

## 5. Conclusions

Based on the findings of this in vitro study, the accuracy of 3D scanners is affected by tooth type. Intraoral scanners show better accuracy in the anterior teeth (central incisor, lateral incisor, and canine) and significantly poor accuracy in the second molars. Thus, the accuracy of intraoral scanners tends to get worse from anterior to posterior. On the other hand, the accuracy of the desktop scanners was influenced by the tooth type, but there were slight differences. Therefore, when scanning a complete arch using an intraoral scanner, the clinician should consider the tendency for the accuracy to decrease from anterior to posterior region. The increased inaccuracy in the posterior region is probably due to the tendency of the complete arch to broaden in the buccal direction with respect to the midline (i500 intraoral scanner tends to narrow towards the lingual direction). This inaccuracy should be overcome by the development of intraoral scanners. Through the advance of medical devices such as intraoral scanner and CBCT scanner in digital workflow, quality of life in patients can be improved.

## Figures and Tables

**Table 1:** Horizontal displacement and 3D displacement according to the 3D scanners.

Displacement Type	Desktop Scanner		Intraoral Scanner					*P*
	3Shape E1	DOF	CS3500	CS3600	Trios2	Trios3	i500	
	RMS (Mean ± SD, μm)							
Horizontal displacement	23.8 ± 0.8^A^	29.4 ± 0.5^AB^	51.2 ± 17.4^B^	46.6 ± 13.7^B^	86.5 ± 23.3^C^	43.4 ± 18.1^B^	104.6 ± 23.5^C^	<0.001 *
3D displacement	14.2 ± 0.4^A^	27.8 ± 0.4^AB^	60.7 ± 14^C^	38.7 ± 10.3^BD^	80.6 ± 19^C^	42.9 ± 13.4^D^	93.8 ± 14.5^E^	<0.001 *

* Different capital letters (row) indicate that the difference between the groups is significant by Tukey’s HSD post hoc testing (*P* < 0.05).

**Table 2:** Comparison of the accuracy of 3D scanners according to the tooth type.

Tooth Type	Desktop Scanner		Intraoral Scanner					*P*
	3Shape E1	DOF	CS3500	CS3600	Trios2	Trios3	i500	
	RMS (Mean ± SD, μm)							
Central incisor	9.1 ± 0.3^Aa^	27.7 ± 0.8^Ba^	31.2 ± 6.6^Ba^	26.5 ± 5.9^Ba^	46.9 ± 12.7^Ca^	30.6 ± 8.5^Ba^	52.2 ± 12.8^Cab^	<0.001 *
Lateral incisor	10.1 ± 0.4^Aa^	27.2 ± 0.6^Bab^	36.4 ± 11.5^Ca^	25.5 ± 5^Ba^	40.5 ± 12.6^Ca^	26.5 ± 6.3^Ba^	41.3 ± 11.3^Ca^	<0.001 *
Canine	19.8 ± 1.3^Ab^	25 ± 0.3^Acd^	39.9 ± 12.6^Ba^	25.9 ± 4.2^Aa^	51.9 ± 6.2^Cab^	23.8 ± 5.6^Aa^	39 ± 10.7^Ba^	<0.001 *
First premolar	20.8 ± 1.6^Ab^	23.5 ± 0.3^ABd^	39.7 ± 12^Ca^	26.3 ± 5.2^ABa^	65 ± 7.9^Eabc^	30.3 ± 10.7^Ba^	51.4 ± 10.5^Dab^	<0.001 *
Second premolar	27.3 ± 2.1^ABc^	23.9 ± 0.3^Ad^	43.5 ± 15.1^Ca^	32 ± 7.3^ABCab^	76.8 ± 11.2^Dbc^	38.5 ± 15.4^BCab^	66.6 ± 14.3^Db^	<0.001 *
First molar	20.6 ± 1^Ab^	25.9 ± 0.4^Abc^	60.7 ± 18^Bb^	41 ± 15.4^ABb^	88 ± 29^Cc^	50.6 ± 25.6^Bb^	100.7 ± 22.2^Cc^	<0.001 *
Second molar	14.5 ± 1^Ad^	34.2 ± 3.5^ABe^	117.3 ± 32.3^Dc^	56.9 ± 21.2^BCc^	127.5 ± 59.4^Dd^	78.4 ± 36.3^Cc^	171 ± 23.4^Ed^	<0.001 *
*P*	<0.001 *	< 0.001 *	<0.001 *	< 0.001 *	<0.001 *	< 0.001 *	<0.001 *	

* Different capital letters (row) and lowercase letters (column) indicate that the differences between the groups are significant by Tukey’s HSD post hoc testing (*P* < 0.05).
